# Comparative efficacy of antitumor necrosis factor agents and tacrolimus in naïve steroid-refractory ulcerative colitis patients

**DOI:** 10.1038/s41598-020-68828-z

**Published:** 2020-07-27

**Authors:** Moto Kitayama, Yuko Akazawa, Daisuke Yoshikawa, Shuntaro Higashi, Tomohito Morisaki, Hidetoshi Oda, Maho Ikeda, Yujiro Nakashima, Maiko Tabuchi, Keiichi Hashiguchi, Kayoko Matsushima, Naoyuki Yamaguchi, Hisayoshi Kondo, Kazuhiko Nakao, Fuminao Takeshima

**Affiliations:** 1 Department of Gastroenterology and Hepatology, Graduate School of Biomedical Science, Nagasaki University, 1-7-1 Sakamoto, Nagasaki City, Nagasaki 852-8501 Japan; 2 Tissue and Histopathology Section, Atomic Bomb Disease Institute, Nagasaki University, Nagasaki City, Japan; 3 Department of Gastroenterology and Hepatology, Sasebo City General Hospital, 9-3 Hirase-cho, Sasebo City, Nagasaki 857-8511 Japan; 4 Department of Gastroenterology, Inoue Hospital, 6-12, Takaramachi, Nagasaki City, Nagasaki 850-0045 Japan; 5 Department of Gastroenterology and Hepatology, National Hospital Organization Ureshino Medical Center, 2436 Ureshino-cho, Ureshino City, Saga 843-0393 Japan; 6 Department of Gastroenterology and Hepatology, Sasebo Chuo Hospital, 15 Yamato-cho, Sasebo City, Nagasaki 857-1195 Japan; 7 Department of Internal Medicine, Juko Memorial Nagasaki Hospital, 6-17, Maruo, Nagasaki City, Nagasaki 852-8004 Japan; 8 Department of Gastroenterology, National Hospital Organization Nagasaki Medical Center, 2-1001-1 Kubara, Ohmura City, Nagasaki 856-8562 Japan; 9 Biostatistics Section, Division of Scientific Data Registry, Atomic Bomb Disease Institute, Nagasaki University, Nagasaki, Japan; 10 Department of Internal Medicine, Nagasaki Prefecture Goto Central Hospital, 205 Yoshikugi, Goto City, Nagasaki 853-0031 Japan

**Keywords:** Colonoscopy, Ulcerative colitis

## Abstract

While retrospective studies have compared the efficacy of anti–tumour necrosis factor (TNF) agents and tacrolimus (TAC) in ulcerative colitis (UC), information regarding first-time use of these agents is limited. The aim of our study was to investigate the short- and long-term efficacy of anti-TNF agents [adalimumab (ADA) and infliximab (IFX)] and TAC in anti-TNF agent- and TAC-naïve steroid-refractory UC patients. We evaluated 150 steroid-refractory UC patients receiving anti-TNF agents (IFX: n = 30, ADA: n = 41) or TAC (n = 79) at eight institutions in Japan. Clinical response rates at 8 weeks were 73.2% and 75.9% while remission rates were 30.1% and 25.3% in the anti-TNF and TAC groups, respectively. Logistic regression analysis showed the male sex and higher C-reactive protein to be independent factors for response to anti-TNF agents and TAC, respectively. Use of TAC was an independent factor for relapse. No differences in response to the treatment or relapse were observed between IFX and ADA. In conclusion, TAC and anti-TNF agents promoted similar short-term effects, but anti-TNF agents ensured better long-term outcomes at first-time treatment of steroid-refractory UC patients.

## Introduction

Ulcerative colitis (UC) is a disabling chronic inflammatory condition of the large intestine of unknown aetiology. Moreover, UC markedly impairs patients' quality of life due to its symptoms such as diarrhoea, bloody stool, abdominal cramps, and faecal urgency^1,2^. Conventional therapeutic options including 5-aminosalicylates as first-line therapy and corticosteroids as second-line therapy, are effective in inducing remission in the majority of UC patients. However, 20–50% of patients are either resistant to or dependent on steroids^3–5^. Additionally, a subset of the patients is not able to *tolerate* steroid therapy due to side effects such as worsening of diabetes, osteoporosis, and high blood pressure.

Third-line therapies for steroid-refractory UC such as anti–tumour necrosis factor (TNF) agents or tacrolimus (TAC) are usually considered at this point. The efficacy of anti-TNF agents has been established in randomized controlled trials (RCTs). Infliximab (IFX) was the first anti-TNF agent to demonstrate efficacy in achieving and maintaining clinical remission and response in moderate-to-severe UC in the ACT1 and ACT2 trials^6,7^. Additionally, the efficacy of adalimumab (ADA) and golimumab for the induction and maintenance of remission was also established in the ULTRA1, ULTRA2, ULTRA3^8–10^, and PURSUIT trials^11,12^. The calcineurin inhibitor, TAC, similar to ciclosporin (CsA), has been reported to exert a more potent immunosuppressive effect with less severe adverse events compared to CsA^13–15^. In clinical practice, ADA as well as TAC have been reported to be effective, especially in anti-TNF agent–naïve patients^16,17^. The efficacy of TAC in achieving steroid-refractory UC clinical remission was also demonstrated by RCTs^18,19^. However, the efficiency of TAC in maintaining remission is largely unknown. Therefore, in Japan, TAC is currently being employed as an induction therapy to be given for 3 months, but not as a maintenance therapy.

To date, there is no RCT comparing the efficacy of anti-TNF agents with TAC, but there are several reports of retrospective observational studies conducted in Japan^20,21–26^, where both IFX and TAC appeared to be equally safe and effective as induction therapy. However, most of these studies have investigated the use of IFX and TAC, and data including ADA are rather limited^21,24^. Further, the majority of these studies included either anti-TNF agent–experienced or TAC-experienced patients. Because the response rate of these agents may vary between first time use and experienced cases, further investigation focusing on either condition are required.

Therefore, the present study aimed to assess the contributing factors for effectiveness, and to compare the safety between anti-TNF agents including ADA, and TAC in anti-TNF agent–naïve and TAC-naïve steroid-refractory UC patients.

## Methods

### Study design and patients

This was a multicentre retrospective observational study of steroid-refractory UC patients receiving anti-TNF agents or TAC between March 2010 and March 2017. A total of eight medical sites including Nagasaki University Hospital and its related facilities were involved. This study was performed in accordance with the ethical guidelines of the Declaration of Helsinki and was reviewed and approved by the Nagasaki University Hospital Ethics Committee (Approval number: 16092630-2) before initiation. Informed consent was obtained in the form of opt-out on the website. Patients who did not provide informed consent were excluded from this study: this opt-out consent method was also approved by Nagasaki University Hospital Ethics Committee.

Patients who did not provide informed consent were excluded from this study. The diagnosis of UC was confirmed according to standardized criteria by prior clinical assessment, endoscopy, and histology. Patients who failed to complete 12 weeks of follow-up due to relocation were excluded from the final analysis.

### Treatment protocol

The IFX was administered at a dose of 5 mg/kg at 0, 2, and 6 weeks and then every 8 weeks thereafter. The ADA was administered at an initial dose of 160 mg and a second dose of 80 mg with a 2-week induction interval. Thereafter, ADA 40 mg was administered every other week. However, IFX or ADA dose intensification was not included in this study because it is not currently approved in Japan. The TAC was administered orally at an initial dose of 0.1 mg/kg/day in two divided doses. The doses of TAC were adjusted to achieve first a blood trough level of 10 to 15 ng/mL until week 2, and then a level of 5 to 10 ng/mL thereafter. At week 12, TAC was discontinued based on the rules provided by the Japanese healthcare system. The attending physicians at each site selected from among these protocols at their discretion. All the treatment protocols were conducted according to evidence-based clinical guidelines for Inflammatory Bowel Disease developed by the Japanese Society of Gastroenterology^27^.

### Data collection

A shared common database was used to collect demographic and clinical data relevant to this study. Data collected at baseline included gender, age, disease duration, disease extension, concomitant medications, C-reactive protein (CRP) level, partial Mayo score (pMS), and Mayo endoscopic score (MES). Disease activity was evaluated using the CRP level, pMS, and MES. The date of and reason for treatment discontinuation, requirement for further rescue therapy, and any adverse events were also recorded.

### Definitions

We defined clinical remission as a pMS less than two points together with a score of zero points in the rectal bleeding section. Clinical response was defined as a decrease in pMS by three or more points from baseline. Relapse was defined as the occurrence of any UC clinical symptoms requiring further rescue therapy. Steroid-refractory UC was defined as either steroid resistance or dependence. Steroid resistance was defined as the absence of a response to an oral or intravenous prednisolone dose of more than 30 mg/day for 1 to 2 weeks. Steroid dependence was defined when prednisolone could not be reduced to less than 10 mg/day without disease recurrence or relapse occurring within 3 months of stopping prednisolone.

### Endpoints

The primary endpoints of the study were rates of clinical remission and response at 8 weeks. The secondary endpoints were cumulative relapse-free rates. Long-term outcomes were evaluated using data from patients who were followed up for more than 6 months after IFX, ADA, or TAC treatment.

### Statistical analysis

Statistical analysis was performed by a statistician (HK). Differences in quantitative parameters between the two groups were assessed with the Wilcoxon rank sum test. Fisher’s exact test was used for analysis of categorical data. Differences in the values between the two time periods for each patient were assessed using the Wilcoxon signed rank test. Logistic regression was employed to estimate odds ratios (ORs) and 95% confidence intervals (CIs) for each factor that contributed to remission or effectiveness of the drugs in 8 weeks. The Cox proportional hazard model with person-days as the underlying metric was employed to estimate hazard ratios (HRs) and 95% CIs of each risk factor for relapse of the UC. All statistical tests were two-sided, and p-values of less than 0.05 were considered to be statistically significant. The calculations were performed using the LOGISTIC and PHREG procedures in the SAS software package (version 9.4; SAS Institute, Inc., Cary, NC, USA).

## Results

### Patient demographics

**Figure 1:**
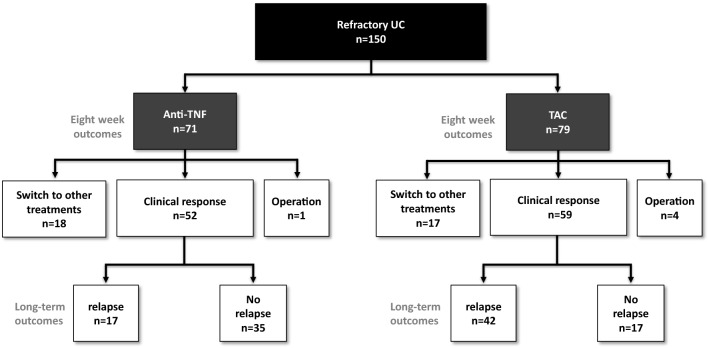
Flowchart of treatment outcomes in the anti-TNF group (n = 71) and the TAC group (n = 79). UC, ulcerative colitis; IFX, infliximab; ADA, adalimumab; TNF, tumour necrosis factor; TAC, tacrolimus.

A total of 150 cases were analysed. Seventy-one individuals were treated with anti-TNF agents, and 79 individuals received TAC (Fig. 1). Baseline characteristics of the 150 study participants are presented in Table 1. Among these patients, the mean age was 46.2 ± 16.7 years, 51.3% were men, the mean disease duration was 90.8 ± 89.9 months, and 64.7% had total colitis. No differences were found with respect to epidemiologic characteristics between the anti-TNF and TAC groups, except for disease extension. The proportion of patients with total colitis, rate of hospitalization, cytomegalovirus (CMV) infection, pMS, MES, and baseline CRP level tended to be higher in the TAC group (Table 1). The rate of steroid-dependent patients with prior treatment consisting of thiopurine was higher in the anti-TNF group, whereas more patients with steroid resistance were found in the TAC group (Table 1).

**Table 1:** Baseline characteristics of patients in the study.

	Total (n = 150)	anti-TNF (n = 71)	TAC (n = 79)	*p* value
**Sex**				
Male, n	77 (51.3%)	32 (45.1%)	45 (57.0%)	0.1457
Female, n	73 (58.7%)	39 (54.9%)	34 (43.0%)	
Age*, median [IQR]	46.5 [32–60.25]	46 [30–61]	47 [32–65]	0.6732
Disease duration (months)*, median [IQR]	61 [24–132]	60 [24–125]	62 [24–132]	0.9280
**Disease extension**				
Left-sided colitis, n	53 (35.3%)	31 (43.7%)	22 (27.8%)	**0.0431**
Total colitis, n	97 (64.7%)	40 (56.3%)	57 (72.2%)	
Hospitalization, n	128 (85.3%)	53 (74.7%)	75 (94.9%)	**0.0005**
**Response to steroid**				
Dependent, n	75 (50.0%)	45 (63.4%)	30 (38.0%)	
Resistant, n	68 (45.3%)	24 (33.8%)	44 (55.7%)	**0.0032**
Intolerant, n	7 (4.7%)	2 (2.8%)	5 (6.3%)	
Thiopurine treatment, n	77 (51.3%)	32 (45.1%)	45 (57.0%)	0.1457
CMV infection, n	29 (19.3%)	7 (9.9%)	22 (27.9%)	**0.0053**
pMS*, mean ± SD	6.54 ± 1.80	5.75 ± 1.72	7.23 ± 1.57	** < 0.0001**
MES*, mean ± SD	2.56 ± 0.65	2.38 ± 0.72	2.71 ± 0.54	**0.0030**
CRP (mg/dL), median [IQR]	1.2 [0.3–3.44]	0.53 [0.235–2.46]	1.8 [0.58–4.87]	**0.0016**

*TNF* tumour necrosis factor, *TAC* tacrolimus, *CMV* cytomegalovirus, *pMS* partial Mayo Score, *MES* Mayo Endoscopic Score, *CRP* C-reactive protein, *SD* standard deviation, *IQR* interquartile range.

Data was analysed either by Fisher's exact test or *Wilcoxon's test.

*p* values less than 0.05 are shown in bold.

Detailed characteristics of the 71 patients who were treated with anti-TNF agents are further shown separately in Table 2. Of these 71 patients, 30 and 41 were treated by IFX and ADA, respectively. No significant differences in the sex ratio, age, disease duration, disease extension, prior treatment with thiopurine, CMV infection, or mean serum CRP level were observed between the IFX and ADA groups. Although most of the patients in the IFX group were hospitalized (96.7%), mostly to monitor infusion reaction, only 61% were hospitalized in the ADA group. Additionally, both the pMS and MES tended to be higher in the IFX than in the ADA group.

**Table 2:** Baseline characteristics of the anti-TNF agent- treated patients.

	IFX (n = 30)	ADA (n = 41)	*p* value
**Sex**			
Male, n	15 (50.0%)	17 (58.5%)	0.4752
Female, n	15 (50.0%)	24 (41.5%)	
Age*, median [IQR]	49.5 [25–61.25]	46 [32–58]	0.9907
Disease Duration (months)*, median [IQR]	55 [22.5–127.75]	60 [27–139.5]	0.5372
**Disease extension**			
Left-sided colitis, n	15 (50.0%)	16 (39.0%)	0.3570
Total colitis, n	15 (50.0%)	25 (61.0%)	
Hospitalization, n	29 (96.7%)	24 (58.5%)	**0.0003**
**Response to steroid**			
Dependent, n	16 (53.3%)	29 (70.7%)	0.0691
Resistant, n	14 (46.7%)	10 (24.4%)	
Intolerant, n	0 (0.0%)	2 (4.9%)	
Thiopurine treatment, n	14 (46.7%)	18 (43.9%)	0.8171
CMV infection, n	3 (10.0%)	4 (9.8%)	0.9728
pMS*, mean ± SD	6.25 ± 1.86	5.4 ± 1.55	0.0549
MES*, mean ± SD	2.58 ± 0.70	2.26 ± 0.72	**0.0417**
CRP (mg/dL), median [IQR]	0.725 [0.3–3.6125]	0.485 [0.105–1.95]	0.2074

*IFX* infliximab, *ADA* adalimumab, *CMV* cytomegalovirus, *pMS* partial Mayo Score, *MES* Mayo Endoscopic Score, *CRP* C-reactive protein, *SD* standard deviation, *IQR* interquartile range.

Data was analysed either by Fisher's exact test or *Wilcoxon's test.

*p *values less than 0.005 are shown in bold.

### Clinical efficacy at 8 weeks

The clinical response rates at 8 weeks after treatment were 74.0% (111/150) in total, and 73.2% (52/71) and 74.7% (59/79) in the anti-TNF and TAC groups, respectively. The clinical remission rates at 8 weeks after the start of treatment were also comparable (28.7% in total, 31.0% and 26.6% in the anti-TNF and TAC groups, respectively). No differences in response rate or remission rate were observed, by Fisher’s exact test, between the TAC and anti-TNF groups.

**Figure 2:**
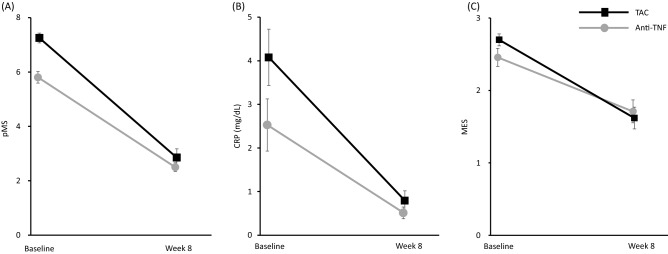
Changes in partial Mayo Score (pMS) (**A**), C-reactive protein(CRP) (**B**), Mayo Endoscopic Score (MES) (**C**) during the 8-week treatment period in the tacrolimus (TAC) and anti-TNF groups. Mean ± standard error values are presented. Statistical differences between baseline versus week 8 are shown: (**A**) *p* < 0.0001, TAC; *p* < 0.0001, Anti-TNF. (**B**) *p* < 0.0001, TAC; *p* = 0.0003, Anti-TNF. (**C**) *p* < 0.0001, TAC; *p* < 0.0001, Anti-TNF.

Changes in the pMS and MES and the CRP level during the 8 week-treatment period are presented in Fig. 2. The corresponding pMS, MES, and CRP level at baseline and 8 weeks after the start of therapy decreased in both the TAC and TNF groups.

Subsequently, the anti-TNF group was analysed separately. The clinical response rates at 8 weeks after treatment were 76.7% (23/30) and 70.7% (29/41) in the IFX and ADA groups, respectively. Remission rates were 13/30 (43.3%) and 9/41 (22.0%) in the IFX and ADA groups, respectively. No differences in response rate or remission rate were observed, by Fisher’s exact test, between the IFX and ADA groups.

**Figure 3:**
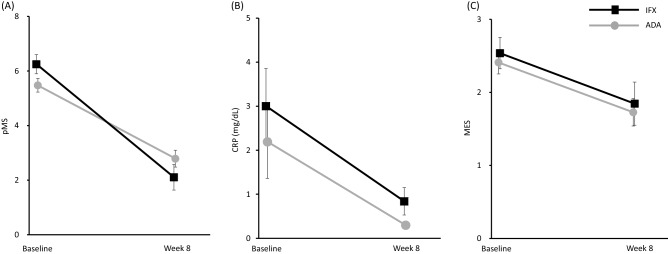
Changes in partial Mayo Score (pMS) (**A**), C-reactive protein (CRP) (**B**), Mayo Endoscopic Score (MES) (**C**) during the 8-week treatment period in the infliximab (IFX) and adalimumab (ADA) groups. Mean ± standard error values are presented. Statistical differences between baseline versus week 8 are shown: (**A**) *p* < 0.0001, IFX; *p* < 0.0001, ADA (**B**) *p* = 0.0011, IFX group; *p* < 0.0001, ADA Group. (**C**) *p* = 0.0313, IFX group; *p* = 0.0103, ADA Group.

The pMS, CRP, and EMS improved significantly at 8 weeks after initiation of the treatments in both the IFX and ADA groups (Fig. 3A-C).

Among the total cases, multivariate logistic regression analysis revealed that the male sex was more likely to respond to the treatment (OR 2.397, 95% CI 1.009–5.690, p = 0.0476) (Table 3). Use of azathioprine and 6-mercaptopurine (AZA/6MP) tended to increase the odds for response, although not reaching statistical difference (*p* = 0.053). Subsequently, factors affecting clinical response were separately investigated, by logistic regression analysis, in the TAC and anti‐TNF groups. In the TAC group, high CRP increased the odds of treatment response (OR: 1.548, 95% CI: 1.031–2.234, *p* = 0.0351). In contrast, the serum CRP before treatment did not have a significant impact on the patients’ response to treatment in the anti-TNF group. Within the anti-TNF group overall, selection of IFX or ADA did not affect the odds of response in 8 weeks. The male sex increased the odds of effectiveness only in the TNF group (OR 4.455, 95% CI 1.099–18.107, *p* = 0.0368).

**Table 3:** Logistic regression analysis of factors affecting clinical response at 8 weeks.

Predictors	Overall n = 150			Anti-TNF n = 71			TAC n = 79		
	OR	95% CI	*p* value	OR	95% CI	*p* value	OR	95% CI	*p* value
TAC	0.748	0.271–2.066	0.5751	–	–	–	–	–	–
ADA	–	–	–	1.400	0.246–7.956	0.7044	–	–	–
Male sex	**2.397**	**1.009–5.690**	**0.0476**	**4.455**	**1.096–18.107**	**0.0368**	1.571	0.430–5.742	0.4944
Age per year	0.997	0.970–1.025	0.8387	1.015	0.969–1.062	0.5369	0.983	0.940–1.025	0.4247
Disease duration per month	1.004	0.998–1.010	0.1846	1.008	0.998–1.019	0.1271	1.002	0.994–1.011	0.5896
Disease extension total	0.976	0.394–2.414	0.9577	0.886	0.218–3.609	0.8659	1.582	0.383–6.524	0.5261
Hospitalization	0.890	0.238–3.325	0.8622	2.339	0.361–15.141	0.3726	< 0.001	< 0.001- > 999.999	0.9785
Steroid resistance	0.792	0.304–2.064	0.6338	1.158	0.231–5.808	0.8582	0.490	0.118–2.030	0.3253
Thiopurine treatment	2.462	0.989–6.132	0.0529	1.817	0.442–7.471	0.4078	2.821	0.685–11.615	0.1509
CMV infection	1.038	0.346–3.111	0.9472	4.116	0.318–53.245	0.2787	0.617	0.145–2.628	0.5135
PMS	0.980	0.746–1.287	0.8848	1.194	0.741–1.924	0.4663	0.863	0.564–1.321	0.4978
MES	0.978	0.470–2.039	0.9535	0.846	0.309–2.321	0.7461	1.123	0.308–4.094	0.8605
Serum CRP level	1.138	0.988–1.310	0.0730	1.005	0.862–1.172	0.9471	**1.548**	**1.031–2.324**	**0.0351**

*TNF* tumour necrosis factor, *TAC* tacrolimus, *ADA* adalimumab, *CMV* cytomegalovirus, *pMS* partial Mayo Score, *MES* Mayo Endoscopic Score, *CRP* C-reactive protein, *OR* odds ratio, *CI* confidence interval.

Factors affecting clinical response were assessed by logistic analysis.

Statistically significant affecting factors are shown in bold.

Other factors including age, hospitalization, and Mayo score at the beginning of the treatment did not seem to significantly contribute to the outcomes of short-term response to either drugs in the logistic regression analysis (Table 3).

### Long-term outcomes

**Figure 4:**
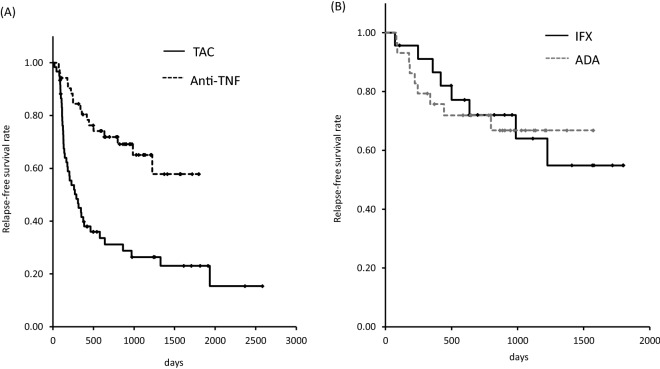
Plots for relapse-free survival in the anti-TNF and TAC (**A**) as well as TNF groups (**B**). TNF, tumour necrosis factor; TAC, tacrolimus.

Long-term outcomes of overall therapy in the multivariate Cox regression models are shown in Table 4. Analysis of overall cases indicated that the use of TAC rather than anti-TNF therapy was an independent risk factor for relapse (HR 4.284, 95% CI 2.143–8.564 *p* < 0.0001). A higher Mayo score at 8 weeks after treatment initiation also significantly increased the risk for relapse in all cases (HR 1.305, 95% CI 1.062–1.603). Other factors such as age, sex, use of AZA/6MP, and steroid resistance were not significantly associated with clinical relapse. Figure 4A demonstrates the relapse free-survival period in the TNF versus TAC groups.

**Table 4:** Cox-hazard model of relapse rate in the patients after responding to the initial treatment.

Predictors	Overall (n = 150)			Anti-TNF (n = 71)			TAC (n = 79)		
	HR	(95% CI)	*p* value	HR	(95% CI)	*p* value	HR	95% (CI)	*p* value
TAC	**4.284**	**(2.143–8.564)**	** < 0.0001**	–	–	–	–	–	–
ADA	–	–	–	0.633	(0.179–2.241)	0.4783	–	–	–
Male sex	1.264	(0.677–2.359)	0.4620	2.091	(0.524–8.345)	0.2960	1.166	(0.540–2.513)	0.6961
Age (per year)	1.013	(0.992–2.359)	0.2125	0.999	(0.961–1.039)	0.9765	1.016	(0.991–1.042)	0.2084
Disease duration (per month)	1.002	(0.997–1.006)	0.4884	1.005	(0.997–1.013)	0.2300	0.999	(0.993–1.005)	0.7292
Disease extension total	0.822	(0.421–1.606)	0.5662	1.953	(0.620–6.155)	0.2528	0.498	(0.209–1.191)	0.1173
Steroid resistance	0.984	(0.530–1.824)	0.9582	1.247	(0.332–4.685)	0.7436	0.841	(0.393–1.798)	0.6556
CMV infection	0.694	(0.303–1.591)	0.3886	0.630	(0.060–6.563)	0.6991	0.811	(0.316–2.079)	0.6621
AZA/6-MP	0.997	(0.514–1.935)	0.9930	2.380	(0.747–7.576)	0.1423	0.610	(0.255–1.460)	0.2672
PMS at 8 weeks	**1.305**	**(1.062–1.603)**	**0.0114**	1.460	(0.970–2.199)	0.0699	1.212	(0.943–1.557)	0.1337
CRP at 8 weeks	1.846	(0.891–3.827)	0.0991	1.377	(0.252–7.524)	0.7118	2.084	(0.752–5.772)	0.1578

*TNF* tumour necrosis factor, *TAC* tacrolimus, *ADA* adalimumab, *CMV* cytomegalovirus, *pMS* partial Mayo Score, *MES* Mayo Endoscopic Score, *CRP* C-reactive protein, *HR* hazard ratio, *CI* confidence interval.

Factors affecting clinical response were assessed by Cox-hazard models.

Statistically significant affecting factors are shown in bold.

Factors affecting clinical response were then investigated separately in the TAC and anti‐TNF groups. However, there were no significant factors that influenced the long-term outcomes in multivariate analysis, suggesting similar drug persistence on both drug choices. The relapse free survival period in the IFX versus ADA groups is shown in Fig. 4B.

### Adverse effects

No deaths occurred during the study period. In the IFX group, IFX was withdrawn in five patients (17%), including three females with infusion reaction, a male with seroconversion of the interferon-gamma release assay (a marker for tuberculosis), and another male with pneumocystis pneumonia. On the other hand, ADA was withdrawn in one female patient (2%) who experienced skin eruption. In the TAC group, TAC was withdrawn in three patients (4%), including two cases with leukocytopenia (one female and one male) and one male with renal dysfunction.

## Discussion

This observational study, to our knowledge, is the largest case series to date comparing the efficacy of anti-TNF agents and TAC in steroid-refractory UC patients in routine clinical practice involving multiple facilities. The features of the present study are the focus on anti-TNF agent–naïve and TAC-naïve steroid-refractory UC patients and the fact that more than half of the participants treated with TNF agents were treated with ADA.

Both TAC and anti-TNF agents yielded sufficient response rates at 8 weeks: our logistic regression analysis showed that the selection of either treatment did not statistically influence the response. This is in line with the observations of Yamamoto et al.^21^, where most patients treated with anti-TNF agents were treated with IFX. In fact, the baseline clinical condition of UC in our study was worse in the TAC compared to the TNF group. These tendencies are observed in other studies as well: it has been demonstrated that physicians tended to select TAC, because of its highly immunosuppressive effect, for use in patients with severe UC^22,25^. Additionally, lower clinical activity has been shown to be associated with better response to anti-TNF agents, but not in patients treated with TAC^21^. Indeed, in our study, higher CRP increased the odds of response within the TAC group, suggesting that in severe steroid-refractory UC, TAC may be an optimal choice for inducing remission.

The current study also showed that the overall OR of treatment effectiveness at 8 weeks was significantly higher in male than in female patients. The odds became higher when the data was analysed within the anti-TNF group, whereas this trend was not observed in the TAC group. The impact of sex in response to TNF inhibitors in UC has been reported previously. For instance, significantly lower response rates to treatment with TNF inhibitors are known in not only inflammatory bowel diseases, but also in ankylosing spondylitis^28^. This may be due to a higher rate of production of the anti-TNF antibody, which decreases the blood concentration of the agent, lowering the effect in females^29^. In addition, more females than males have been reported to discontinue ADA due to adverse events, especially skin reactions^30^. In fact, only female patients experienced an infusion reaction and skin eruption in this study. Our study and others show that attention is required for a potentially lower response and more adverse events in females undergoing anti-TNF agent treatment. Considering the data for the IFX and ADA subgroups, we did not see any differences in overall efficiency in 8 weeks. Mizoshita et al.^31^ showed that the efficacy of ADA in remission induction was also equivalent to that of IFX in UC patients who had not previously used anti-TNF agents. Contrarily, a recent indirect comparison meta-analysis^32^ and network meta-analysis^33,34^ showed that IFX is superior to ADA in the induction of remission in UC patients. Although further study is required, our data suggest that steroid-refractory UC in anti-TNF agent naïve cases responds well to both agents, and drug choice can be made considering the patient’s condition. Our Cox hazard model showed that the use of TAC increased the odds of relapse compared to the anti-TNF therapy. Based on the 3 month-prescription rule of TAC in Japan, in most of the studies with long-term outcomes of TAC, TAC would have been switched to thiopurine at 3 months^22,24,26^. In contrast, anti-TNF therapy could go on while the treatment is effective without adverse side effects. Under these conditions, it is understandable that anti-TNF therapy is better than TAC treatment for attaining remission. Although some patients maintain long-term remission after introducing TAC, other treatment options should be prepared when patients show signs of relapse. Optimal alternative treatments would include anti-TNF agents, *ustekinumab,* vedolizumab, and tofacitiniby^35,36^. This study has several limitations. First, it included unselected patients with heterogeneous baseline characteristics in a real practice setting, which was adjusted by multivariable analysis. Head-to- head comparison in a large population with long-term TAC administration in a prospective manner is needed. Second, we did not evaluate the trough level of either IFX or ADA nor the faecal calprotectin levels: monitoring of these was not approved in Japan during the research period.

In conclusion, our results indicated that TAC and anti-TNF agents exert similar satisfactory short-term effects. However, anti-TNF agents yielded better long-term outcomes than TAC in the treatment of TAC- and anti-TNF agent–naïve steroid-refractory UC patients.

## Data Availability

All data generated or analysed in this study are included in this published article.
